# Parasitemia Levels in Trypanosoma cruzi Infection in Spain, an Area Where the Disease Is Not Endemic: Trends by Different Molecular Approaches

**DOI:** 10.1128/spectrum.02628-22

**Published:** 2022-10-03

**Authors:** Maria D. Flores-Chavez, Alba Abras, Cristina Ballart, Ismael Ibáñez-Perez, Pilar Perez-Gordillo, Montserrat Gállego, Carmen Muñoz, Zaira Moure, Elena Sulleiro, Javier Nieto, Emilia García Diez, Lorena Simón, Israel Cruz, Albert Picado

**Affiliations:** a National Centre for Microbiology, Instituto de Salud Carlos III, Madrid, Spain; b Fundación Mundo Sano-España, Madrid, Spain; c Departament de Biologia, Universitat de Girona, Girona, Spain; d Secció de Parasitologia, Departament de Biologia, Sanitat i Medi Ambient, Facultat de Farmàcia i Ciències de l’Alimentació, Universitat de Barcelona, Barcelona, Spain; e ISGlobal, Barcelona, Hospital Clínic, Universitat de Barcelona, Barcelona, Spain; f Centro de Investigación Biomédica en Red de Enfermedades Infecciosas, Instituto de Salud Carlos III (CIBERINFEC, ISCIII), Madrid, Spain; g Servei de Microbiologia, Hospital de la Santa Creu i Sant Pau, Barcelona, Spain; h Institut de Recerca Biomèdica Sant Pau, Barcelona, Spain; i Departament de Genètica i Microbiologia, Universitat Autònoma de Barcelona, Cerdanyola del Vallès, Spain; j Servicio de Microbiología, Hospital Universitario Marqués de Valdecilla, Santander, Spain; k Microbiology Department, Vall d’Hebron Hospital, PROSICS Barcelona, Universitat Autònoma de Barcelona, Barcelona, Spain; l National Centre of Epidemiology, Instituto de Salud Carlos III, Madrid, Spain; m National School of Public Health, Instituto de Salud Carlos III, Madrid, Spain; n Foundation for Innovative New Diagnostics (FIND), Geneva, Switzerland; National Institutes of Health

**Keywords:** Chagas disease, LAMP, *Trypanosoma cruzi*, acute reactivation, chronic infection, congenital infection, molecular diagnosis, parasite load, parasitemia quantification, qPCR

## Abstract

Trypanosoma cruzi infection has expanded globally through human migration. In Spain, the mother-to-child route is the mode of transmission contributing to autochthonous Chagas disease (CD); however, most people acquired the infection in their country of origin and were diagnosed in the chronic phase (imported chronic CD). In this context, we assessed the quantitative potential of the Loopamp Trypanosoma cruzi detection kit (Sat-TcLAMP) based on satellite DNA (Sat-DNA) to determine parasitemia levels compared to those detected by real-time quantitative PCRs (qPCRs) targeting Sat-DNA (Sat-qPCR) and kinetoplast DNA minicircles (kDNA-qPCR). This study included 173 specimens from 39 autochthonous congenital and 116 imported chronic CD cases diagnosed in Spain. kDNA-qPCR showed higher sensitivity than Sat-qPCR and Sat-TcLAMP. According to all quantitative approaches, parasitemia levels were significantly higher in congenital infection than in chronic CD (1 × 10^−1^ to 5 × 10^5^ versus >1 × 10^−1^ to 6 × 10^3^ parasite equivalents/mL, respectively [*P* < 0.001]). Sat-TcLAMP, Sat-qPCR, and kDNA-qPCR results were equivalent at high levels of parasitemia (*P* = 0.381). Discrepancies were significant for low levels of parasitemia and older individuals. Differences between Sat-TcLAMP and Sat-qPCR were not qualitatively significant, but estimations of parasitemia using Sat-TcLAMP were closer to those by kDNA-qPCR. Parasitemia changes were assessed in 6 individual cases in follow-up, in which trends showed similar patterns by all quantitative approaches. At high levels of parasitemia, Sat-TcLAMP, Sat-qPCR, and kDNA-qPCR worked similarly, but significant differences were found for the low levels characteristic of late chronic CD. A suitable harmonization strategy needs to be developed for low-level parasitemia detection using Sat-DNA- and kDNA-based tests.

**IMPORTANCE** Currently, molecular equipment has been introduced into many health care centers, even in low-income countries. PCR, qPCR, and loop-mediated isothermal amplification (LAMP) are becoming more accessible for the diagnosis of neglected infectious diseases. Chagas disease (CD) is spreading worldwide, and in countries where the disease is not endemic, such as Spain, the parasite Trypanosoma cruzi is transmitted from mother to child (congenital CD). Here, we explore why LAMP, aimed at detecting T. cruzi parasite DNA, is a reliable option for the diagnosis of congenital CD and the early detection of reactivation in chronic infection. When the parasite load is high, LAMP is equivalent to any qPCR. In addition, the estimations of T. cruzi parasitemia in patients living in Spain, a country where the disease is not endemic, resemble natural evolution in areas of endemicity. If molecular tests are introduced into the diagnostic algorithm for congenital infection, early diagnosis and timely treatment would be accomplished, so the interruption of vertical transmission can be an achievable goal.

## INTRODUCTION

Chagas disease (CD), or Trypanosoma cruzi infection, has expanded globally through migrants from areas where the disease is endemic, resulting in an increasing public health concern in countries where the disease is not endemic. At present, half of the 4.6 million Latin American migrants who have settled in Europe live in Spain (1). In countries where the disease is not endemic, transmission of T. cruzi not mediated by its biological vector occurs from mother to child during pregnancy, by the transfusion of blood products, or by organ transplantation (2).

In Spain, both transfusion and transplant transmission are routes that are rigorously controlled by the serological screening of donors and the close monitoring of recipients of organs from chagasic donors (3, 4); therefore, the mother-to-child route is the main mode of transmission contributing to autochthonous CD. Although there is as yet no official management plan at the national level (5), serological screening in pregnant women is performed in most hospitals. In areas where the disease is not endemic, vertical transmission is the leading cause of episodes equivalent to acute infection if CD is confirmed in the first months of a child’s life. Without treatment, the acute phase lasts 6 to 8 weeks, and the chronic phase starts when the parasitemia levels decrease and both general symptoms and clinical manifestations, if any, disappear (6). In Spain, the infection is generally detected in the chronic phase (2), but most cases acquired the infection in their country of origin (imported CD).

For diagnosis, parasitological tests are recommended for acute and congenital infection (before the first or second month of the baby’s life), whereas serological tests are used to confirm chronic infection (6, 7). In Spain, PCR and its variant real-time PCR (quantitative PCR [qPCR]) are the diagnostic tools of choice to confirm congenital infection, monitor therapeutic failure, and detect the reactivation of a chronic infection in immunosuppressed patients (8, 9). PCR/qPCR assays have a higher sensitivity than traditional parasitological tests for the detection of the presence of parasites in blood because their limit of detection (LOD) is orders of magnitude lower than those of, e.g., microscopy. PCR can detect a single parasite in 1 mL of blood, whereas reaching this level by direct microscopy requires the exhaustive screening of hundreds of blood films. In addition, PCR is not influenced by the operator’s skills (10, 11).

In recent years, loop-mediated isothermal amplification (LAMP) has emerged as an alternative molecular diagnostic test to PCR (12, 13). This technique amplifies a specific target in a shorter time than PCR/qPCR, under isothermal conditions, and without specific instrumentation (12). Moreover, LAMP results can be interpreted by simple visual examination (12–14), thus simplifying the procedure. In contrast, most LAMP assays are simple tests lacking multiplexing capabilities, which prevents the inclusion of controls to monitor quality control as they cannot be monitored in the same tube. However, currently, LAMP technology is a less expensive alternative to PCR/qPCR.

In previous work, we demonstrated the sensitivity and specificity of the Loopamp Trypanosoma cruzi detection kit (Sat-TcLAMP) (Eiken Chemical Co., Ltd., Japan) for the diagnosis of congenital infection and the detection of parasitemia in chronic infection (14). This kit is a ready-to-use system targeting the highly repetitive satellite sequence of T. cruzi (Sat-DNA) that consists of microtubes containing all of the required reagents dried in their caps. The detection of the amplified products is based on fluorescence due to the calcein contained in the reagent. Fluorescence emission indicates the presence of Sat-DNA of T. cruzi, and it can be detected by visual examination or by using a fluorimeter. Next, Sat-TcLAMP results can be expressed in positive/negative terms and in time-to-positivity (*T_p_*) values. As Sat-TcLAMP was not used previously for quantification, we aimed to assess its potential to determine parasitemia levels compared to qPCRs, one targeting Sat-DNA (Sat-qPCR) and the other based on kinetoplast DNA minicircles (kDNA-qPCR). In this study, we took advantage of our previously reported raw data (14) but reanalyzed them again in quantitative terms together with new data from kDNA-qPCR. In addition, data related to the follow-up of patients in particular situations have also been included. Currently, estimation of a patient’s parasitemia is not used for the routine diagnosis of CD, but in some situations, e.g., when CD and cancer are diagnosed at the same time, the treatment of the cancer is a priority, and the treatment of CD must be delayed, so it is crucial to monitor parasitemia levels to change management decisions. Therefore, we also aimed to understand the variability in estimations of parasitemia using different molecular approaches.

## RESULTS

### Qualitative comparison.

**FIG 1:**
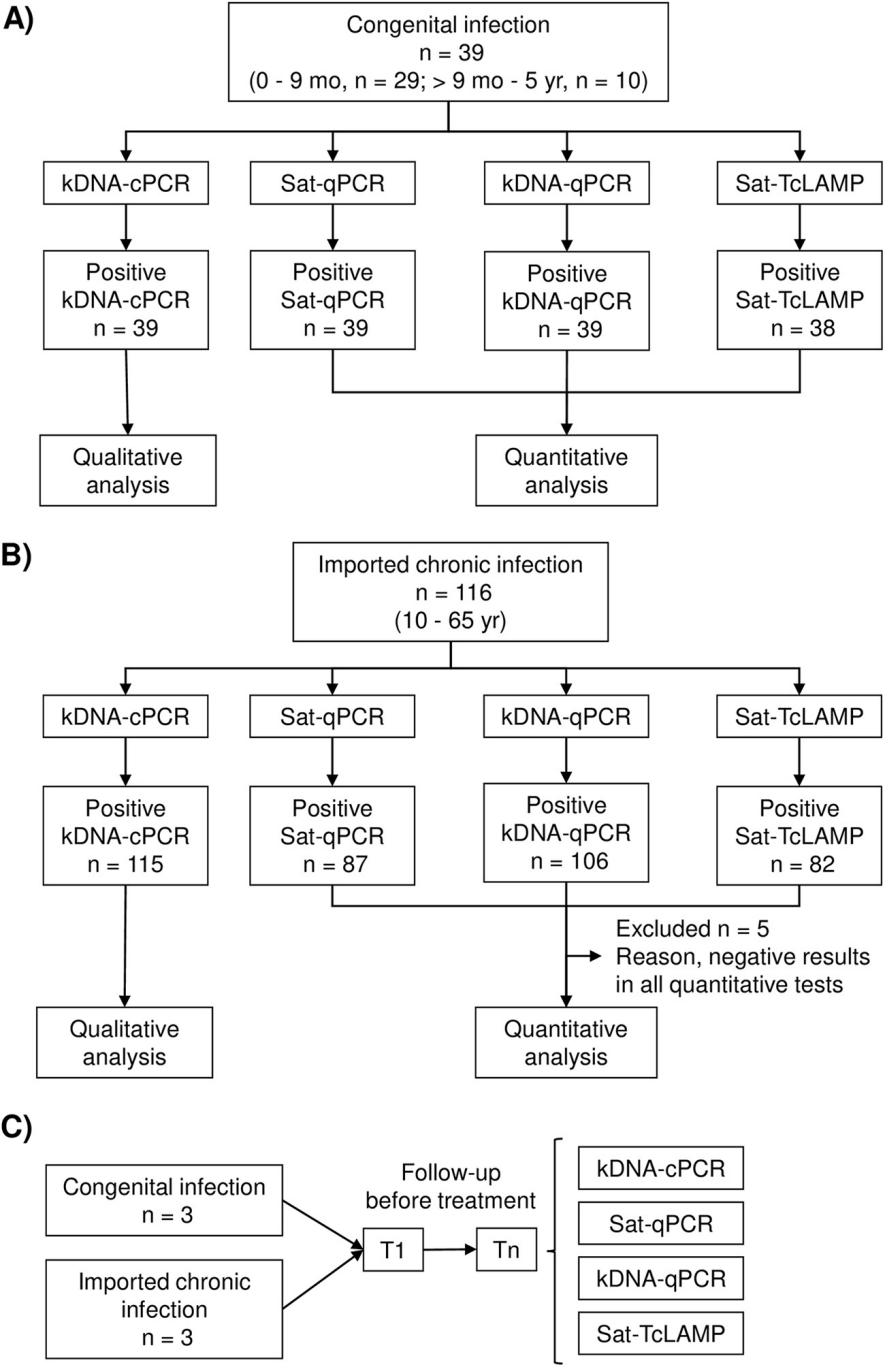
Flow of sample analysis. (A) Samples from congenital infections. (B) Samples from imported chronic infections. (C) Samples from individual cases in follow-up to monitor changes in parasitemia levels before treatment. *T*_1_, time of first sample collection; *T_n_*, time of consecutive sample collection, in one case *T*_2_; in two cases *T*_2_ and *T*_3_; in two other cases *T*_2_, *T*_3_, and *T*_4_; and in the last case *T*_2_ up to *T*_7_.

**FIG 2:**
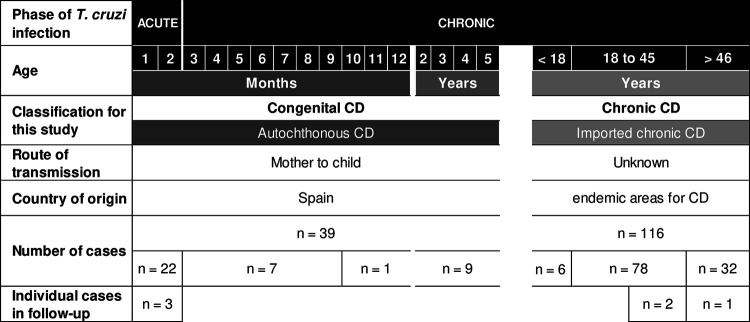
Distribution of cases according to the phase of Chagas disease (CD) and other characteristics.

**FIG 3:**
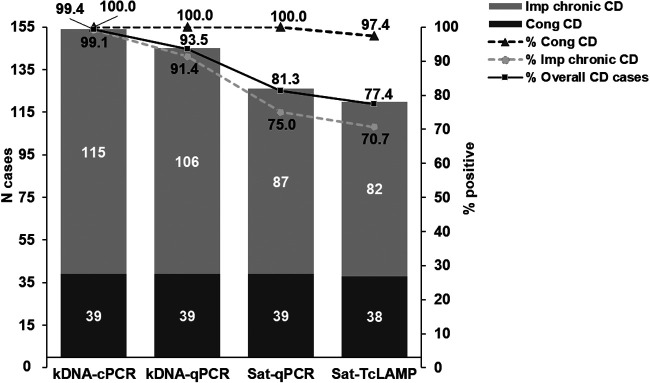
Comparison of the absolute and relative rates of positivity between kDNA and Sat-DNA tests. The rates of positivity in congenital (Cong) infection were similar (*Q* = 3 [*P* = 0.392]), whereas the differences in imported (Imp) chronic infection were significant (*Q* = 57.5 [*P* < 0.001]); there were no significant differences between Sat-DNA tests (*Q* = −0.43 [*P* = 0.321]) and kDNA tests (*Q* = −0.78 [*P* = 0.74]). CD, Chagas disease. The number of cases is indicated in white in each column, and the percentages of positivity for each test are indicated in black.

The flow of sample analysis and the distribution of cases according to the phase of CD and other characteristics are shown in Fig. 1 and 2, respectively. Out of the 173 samples, 171 (98.8%) tested positive by conventional PCR targeting kDNA (kDNA-cPCR), 162 (93.6%) tested positive by kDNA-qPCR, 143 (82.7%) tested positive by Sat-qPCR, and 137 (79.2%) tested positive by Sat-TcLAMP. Considering CD conditions, 155 cases were included in the qualitative comparison, 39 with congenital CD and 116 with chronic CD (Fig. 3). Next, in qualitative terms, 154 (99.4%) patients tested positive by kDNA-cPCR, 145 (93.5%) tested positive by kDNA-qPCR, 126 (81.3%) tested positive by Sat-qPCR, and 120 (77.4%) tested positive by Sat-TcLAMP. Overall, these differences were significant (*Q *= 58.9 [*P* < 0.001]), but pairwise comparisons showed no significant differences between kDNA-cPCR and kDNA-qPCR (*Q *= 0.058 [*P* = 0.077]) and between Sat-qPCR and Sat-TcLAMP (*Q* = −0.39 [*P* = 0.238]). Discrepancies were more frequent between samples from patients with imported chronic infection (*Q* = 57.5 [*P* < 0.001]). The rates of positivity in congenital infection were similar, between 97% and 100% (*Q* = 3 [*P* = 0.392]). On the contrary, for imported chronic infection, the rates of positivity of Sat-DNA tests ranged from 70.7% (82/116) to 75% (87/116), and those of kDNA tests ranged between 914% (106/116) and 99.1% (115/116).

### Comparison of *T_p_* and *C_T_* values.

**FIG 4:**
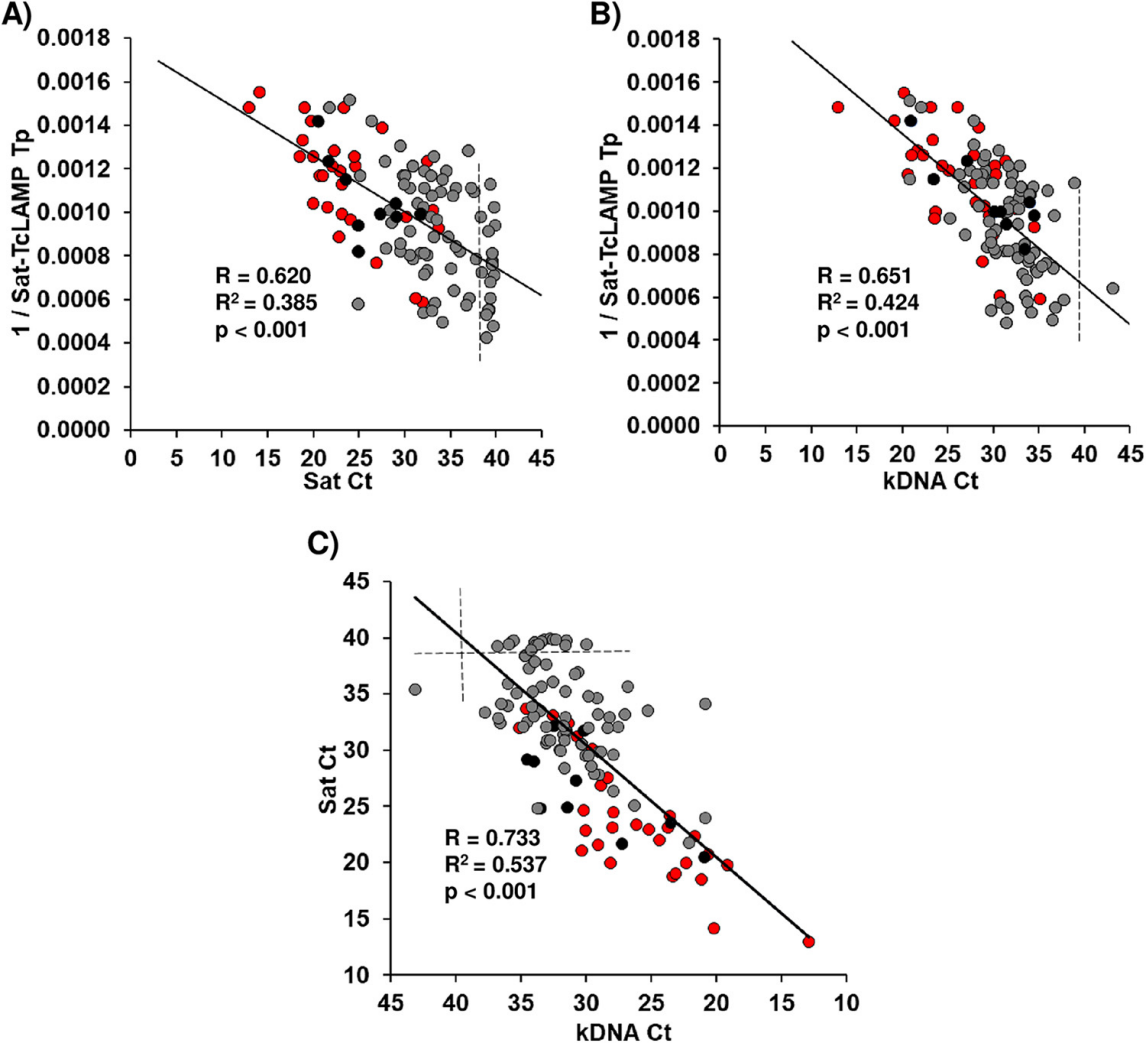
Relationship between *C_T_* and time-to-positivity (*T_p_*) values. (A) Sat-TcLAMP versus Sat-qPCR. (B) Sat-TcLAMP versus kDNA-qPCR. (C) kDNA-qPCR versus Sat-qPCR. The *T_p_* of Sat-TcLAMP is expressed as the inverse to improve the linear shape of the association. Red dots, congenital infection cases diagnosed before 9 months of age; black dots, children with congenital infection diagnosed at between 1 and 5 years of age; gray dots, imported chronic infection cases diagnosed at between 10 and 65 years of age. Dashed lines highlight cases with *C_T_* values outside the quantification range. *R*, coefficient of regression.

Considering the *T_p_* and cycle threshold (*C_T_*) values for all cases (Fig. 4), the linear shape of scatterplots indicated that the association of Sat-TcLAMP versus kDNA-qPCR (*R*^2^ = 0.424) was stronger than that of Sat-TcLAMP versus Sat-qPCR (*R*^2^ = 0.385). Assessing data by Spearman’s rho test, the strength of the association between *T_p_* and *C_T_* values was moderate (*r_s_* = 0.593 [*P* < 0.001] for Sat-TcLAMP *T_p_* versus Sat-qPCR *C_T_*; *r_s_* = 0.644 [*P* < 0.001] for Sat-TcLAMP *T_p_* versus kDNA-qPCR *C_T_*) (see Table S1 in the supplemental material). However, analyzing the data by condition, congenital versus imported chronic infection, we observed a stronger association for congenital infection than for imported chronic infection.

### Standard curve for Sat-TcLAMP and parasite load estimations.

**FIG 5:**
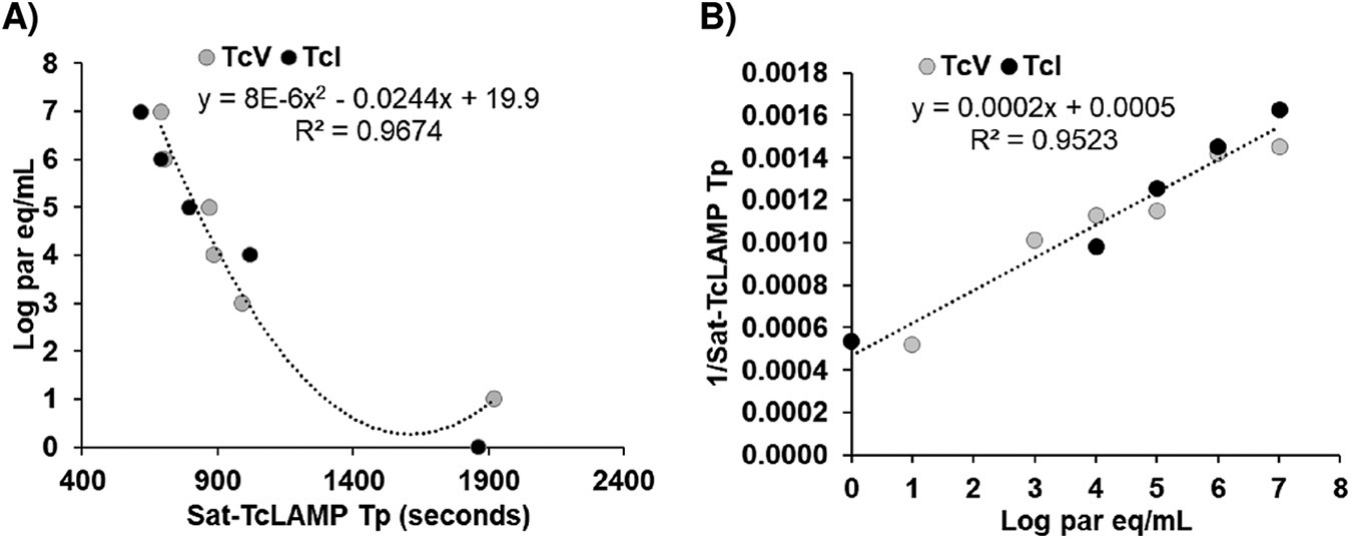
Standard curve for Sat-TcLAMP. Shown are polynomial (A) and linear (B) regressions between the time to positivity (*T_p_*) and parasite equivalents (par eq) (*P* < 0.0001 for both models).

At a high concentration of parasite equivalents (Fig. 5), similar values for *T_p_* were obtained for both T. cruzi discrete typing units (DTUs) TcI and TcV. In contrast, at a low concentration of parasite equivalents, discrepancies were observed (Fig. 5A). This experiment was carried out to compare the analytical sensitivities of Sat-TcLAMP in qualitative terms, but considering the results, the standard curve was built using all data available for both T. cruzi DTUs (Fig. 5B).

Using the equation *y* = 0.0002*x* + 0.0005, with *R*^2^ equal to 95% and a *P* value of <0.0001 (Fig. 5B), the inverse of *T_p_* was extrapolated in parasite equivalents per milliliter assuming the same equivalences as those of qPCRs, with 100 fg DNA as the equivalent to 1 parasite (15). The estimations showed that the parasitemia levels determined using Sat-TcLAMP fluctuated within the range of 4.2 × 10^−1^ to 1.8 × 10^5^ parasite equivalents/mL.

### Comparison of estimations of parasitemia levels.

**FIG 6:**
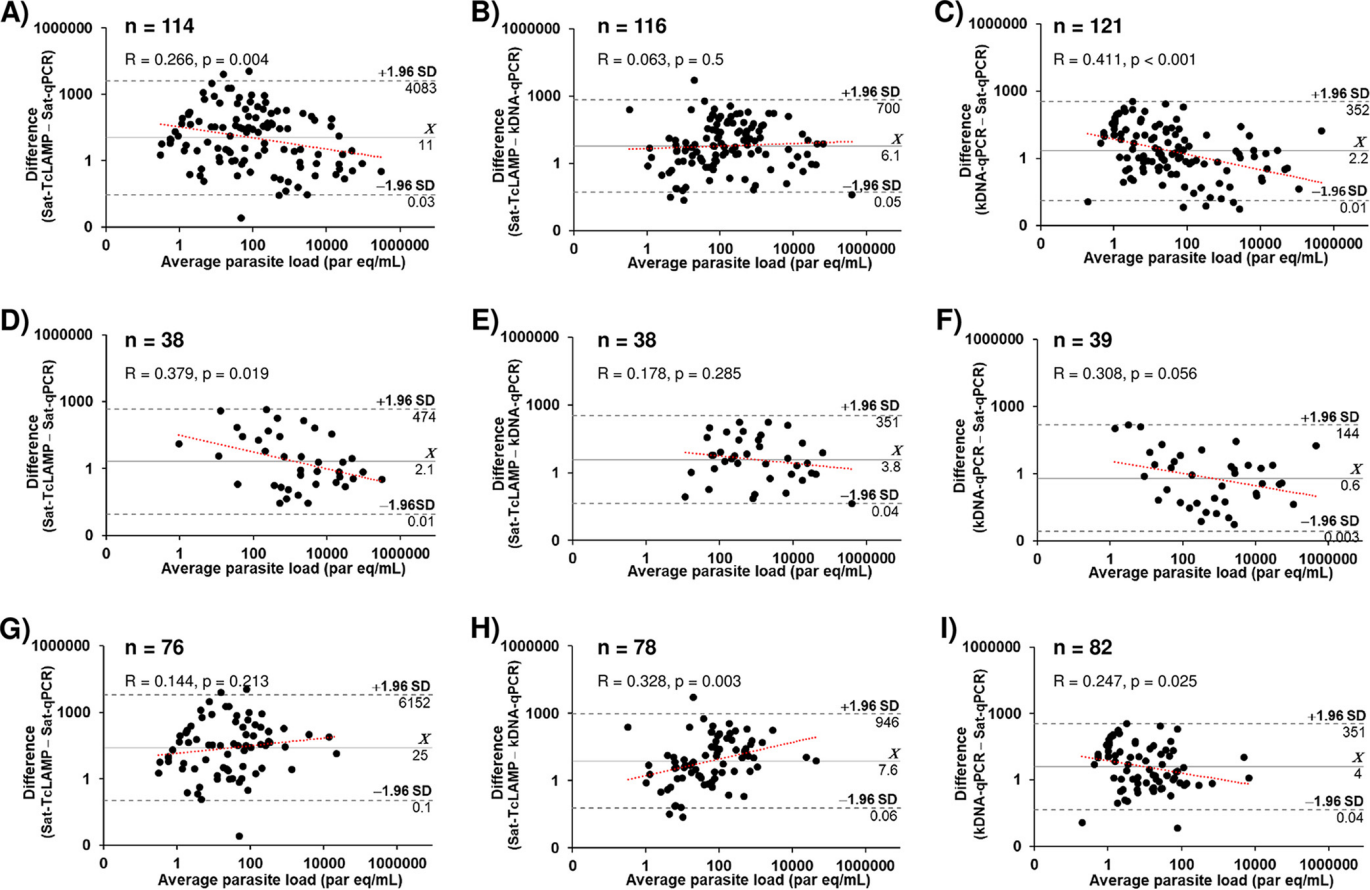
Bland-Altman plots for agreement analysis of parasitemia level estimations by Sat-TcLAMP, Sat-qPCR, and kDNA-qPCR. (A to C) All cases with results by both comparison tests. (D to F) Congenital infection group. (G to I) Imported chronic infection group. The black dashed lines represent the limits of agreement. The red dashed lines represent the linear regression of data points. *Χ*, mean difference (bias regarding difference values of 1); SD, standard deviation; *R*, coefficient of regression; par eq, parasite equivalents. Axes are on logarithmic scales.

**FIG 7:**
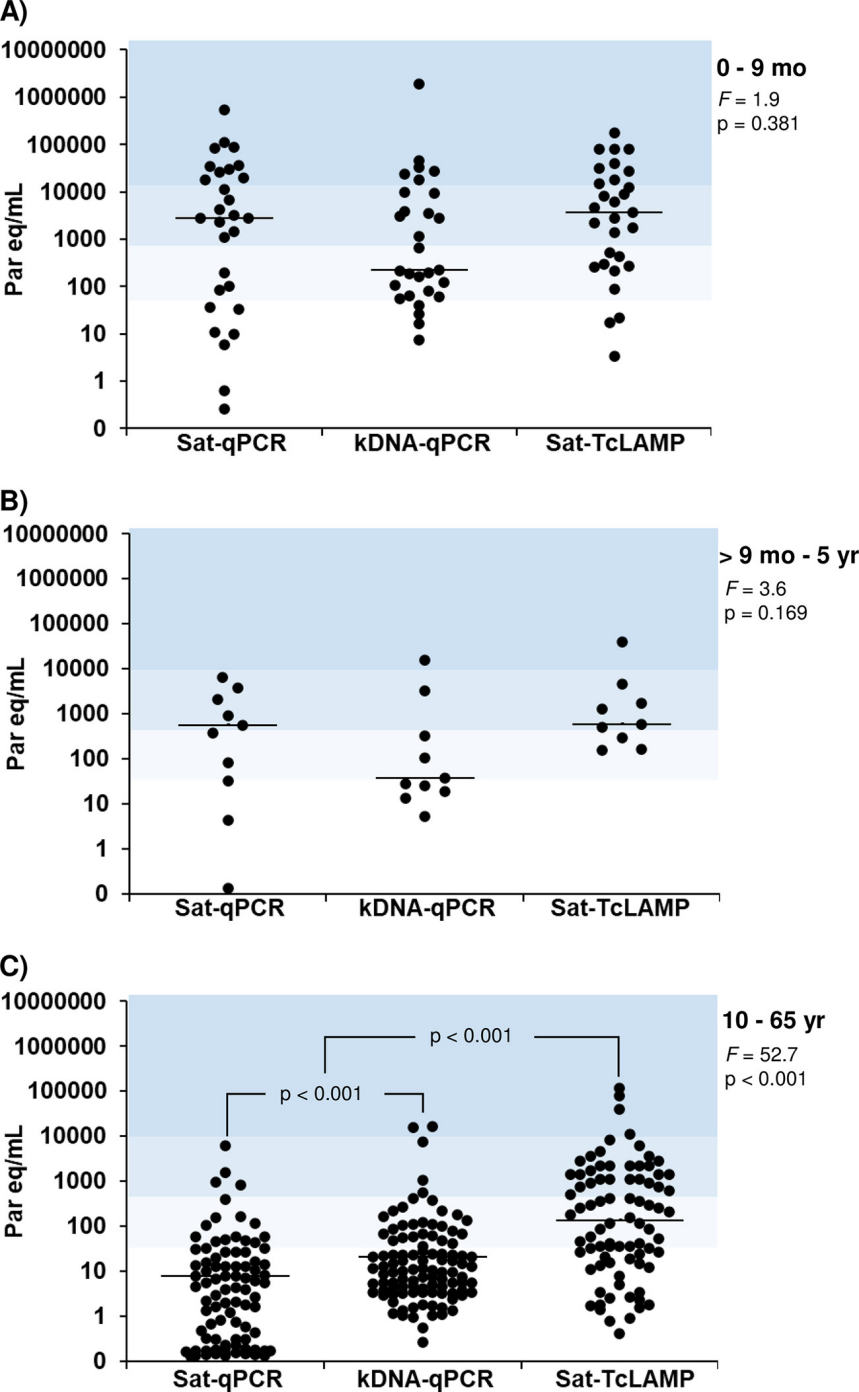
Estimation of parasite loads by Sat-qPCR, kDNA-qPCR, and Sat-TcLAMP. (A) Cases of congenital infection diagnosed before 9 months of age. (B) Cases of congenital infection diagnosed after 9 months and before 5 years of age. (C) Cases of imported chronic infection. The black lines in the dots represent the median values of parasitemia levels. The color background is defined considering the limit of detection of microhematocrit, 0 to 40 parasite equivalents/mL (white), >40 to 500 parasite equivalents/mL (very light blue), >500 to 10,000 parasite equivalents/mL (light blue), and >10,000 parasite equivalents/mL (blue) (31–33). *F*, Friedman test value. The vertical axis is on a semilogarithmic scale.

Sat-qPCR, Sat-TcLAMP, and kDNA-qPCR estimations showed that the parasitemia levels in congenital infection were higher than those in imported chronic infection (all *P* < 0.001) (Table 1). Considering, e.g., Sat-qPCR, the parasite load in congenital cases ranged from 1 × 10^−1^ to 5 × 10^5^ parasite equivalents per mL of blood, whereas in imported chronic CD cases, the range was from 1 × 10^−1^ to 6 × 10^3^ parasite equivalents/mL (Kruskal-Wallis test value = 43.5 [*P* < 0.001]). The pairwise comparisons by Bland-Altman plot analysis (Fig. 6) showed that Sat-TcLAMP estimations had better agreement with kDNA-qPCR estimations than with Sat-qPCR estimations (Fig. 6B and E), although in imported chronic infections, the agreement was better between Sat-TcLAMP and Sat-qPCR values (Fig. 6G). Biases are seen at both the extreme low and high levels of parasitemia. Differences between estimations were assessed by analyzing data from 111 cases that were positive by all molecular tests (Table S2); the distribution of parasitemia levels by Sat-TcLAMP was not equivalent to the distribution by qPCRs (Friedman test value = 44.2 [*P* < 0.001]) (Table S2). However, pondering data according to CD condition (Fig. 7), the quantitative estimations yielded similar distributions of parasite loads in congenital infection, mainly when these cases tested positive before 9 months of age (Friedman test value = 1.9 [*P* = 0.381]) (Fig. 7A). Among the imported chronic cases, 72 out of 116 tested positive by all quantitative tests, and the distributions of parasite loads by each method were significantly different (Friedman test value = 52.7 [*P* < 0.001]) (Fig. 7C).

**TABLE 1:** Comparison of parasite loads considering Chagas disease conditions^*a*^

Parameter	Value for age group				Kruskal-Wallis value (*P* value)
	All cases (0 to 65 yr)	Congenital infection		Imported chronic infection (10 to 65 yr)	
		0 to 9 mo	>9 mo to <5 yr		
Sat-qPCR					43.5 (<0.001)
No. of cases	126	29	10	87	
Parasite equivalents/mL					
Median (*Q*_1_ to *Q*_3_)	12 (1 to 2 × 10^2^)	3 × 10^3^ (84 to 3 × 10^4^)	474 (32 to 2 × 10^3^)	4 (0.3 to 26)	
Min to max	0.1 to 5 × 10^5^	0.3 to 5 × 10^5^	0.1 to 6 × 10^3^	0.1 to 6 × 10^3^	
kDNA-qPCR					44.7 (<0.001)
No. of cases	145	29	10	106	
Parasite equivalents/mL					
Median (*Q*_1_ to *Q*_3_)	22 (5 to 1 × 10^2^)	2 × 10^2^ (80 to 9 × 10^3^)	33 (19 to 3 × 10^2^)	10 (3 to 49)	
Min to max	0.02 to 2 × 10^6^	8 to 2 × 10^6^	5 to 2 × 10^4^	0.02 to 2 × 10^4^	
Sat-TcLAMP					19.7 (<0.001)
No. of cases	120	29	9	82	
Parasite equivalents/mL					
Median (*Q*_1_ to *Q*_3_)	390 (35 to 2 × 10^3^)	4.10^3^ (299 to 2 × 10^4^)	597 (299 to 2 × 10^3^)	170 (19 to 1 × 10^3^)	
Min to max	0.4 to 2 × 10^5^	3 to 2 × 10^5^	157 to 4 × 10^4^	0.4 to 1 × 10^5^	

a The null hypothesis that the distributions of the parasite loads measured by Sat-qPCR, kDNA-qPCR, or Sat-TcLAMP were the same between the different CD conditions was rejected for all molecular approaches. The parasite load is expressed as parasite equivalents per milliliter of blood.

### Comparison of parasitemia trends by age.

**FIG 8:**
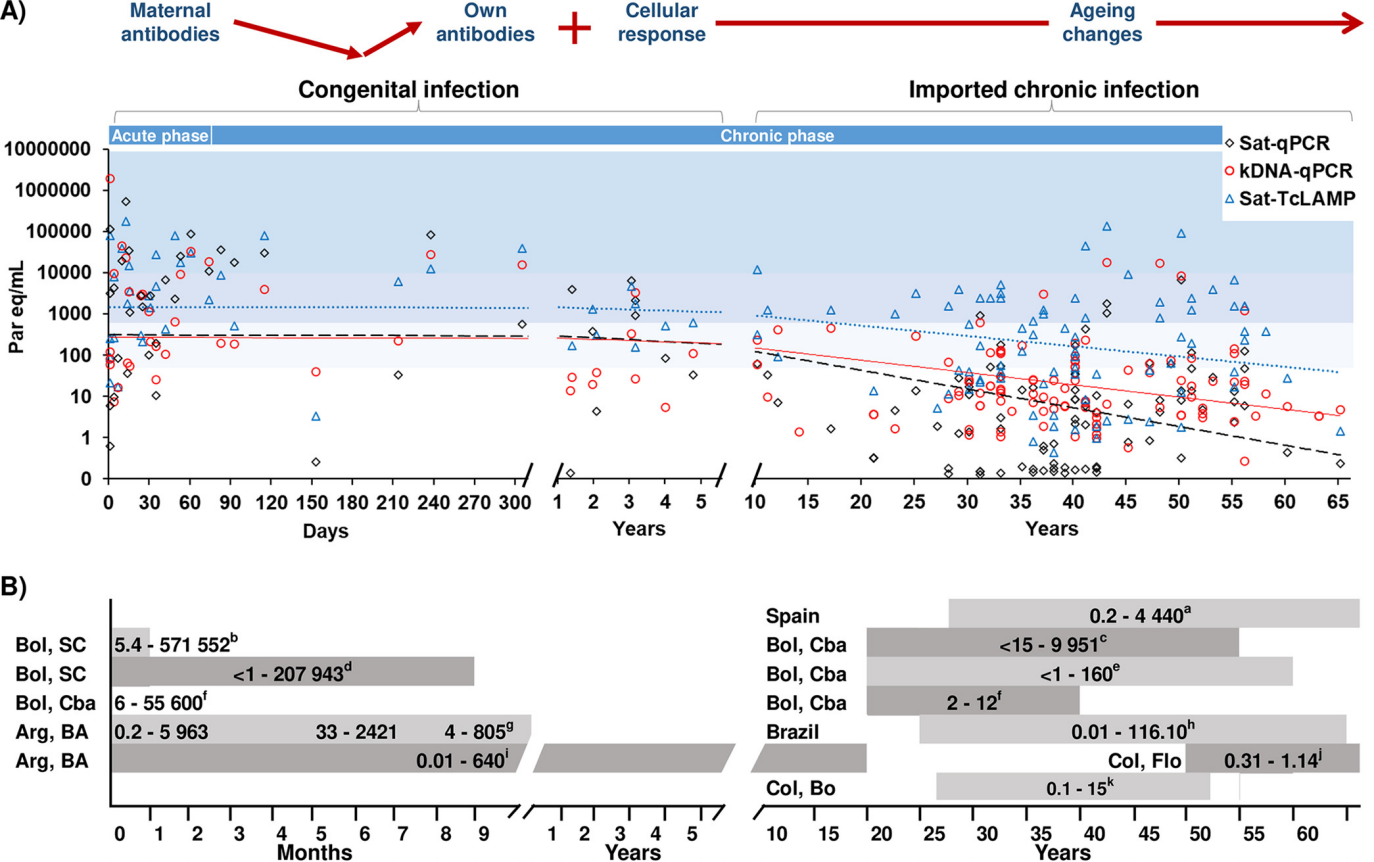
Parasite loads according to age at diagnosis. (A) Data interpretation from this study. (B) Parasitemia levels from other studies. Values are in parasite equivalents (par eq) per milliliter. According to our data, the strength of the association between parasitemia levels and age was moderate from 0 to 38 years of age (Sat-TcLAMP versus age, *r_s_* = −0.456 [*P* < 0.001]; Sat-qPCR versus age, *r_s_* = −0.647 [*P* < 0.001]; kDNA-qPCR versus age, *r_s_* = −0.583 [*P* < 0.001]). ^a^, reference 21; ^b^, reference 11; ^c^, reference 46; ^d^, reference 34; ^e^, reference 47; ^f^, reference 24; ^g^, reference 35; ^h^, reference 17; ^i^, reference 20; ^j^, reference 48; ^k^, reference 38. Bol, Bolivia; SC, Santa Cruz; Cba, Cochabamba; Arg, Argentina; BA, Buenos Aires; Col, Colombia; Flo, Florinda; Bo, Bogota. Dashed lines are trend lines. The color background is defined considering the limit of detection of microhematocrit, 0 to 40 parasite equivalents/mL (white), >40 to 500 parasite equivalents/mL (very light blue), >500 to 10,000 parasite equivalents/mL (light blue), and >10,000 parasite equivalents/mL (blue) (31–33).

Considering the data independently, both Sat-TcLAMP and qPCR showed that the parasitemia levels decreased as infected individuals grew older (Fig. 8). The negative association was weak with respect to Sat-TcLAMP and Sat-qPCR and moderate with respect to kDNA-qPCR (age versus Sat-TcLAMP [*n* = 120], *r_s_* = −0.329 [*P* < 0.001]; age versus Sat-qPCR [*n* = 126], *r_s_* = −0.392 [*P* < 0.001]; age versus kDNA-qPCR [*n* = 145], *r_s_* = −0.466 [*P* < 0.001]). However, according to the regression linear model, the parasite load by Sat-qPCR was related to the age of the patient (*P* < 0.05), while those by kDNA-qPCR and Sat-TcLAMP were not. Reanalyzing the data considering the regression linear model results, the association of parasite load and age was stronger from 0 to 38 years of age (Sat-TcLAMP versus age [*n* = 78], *r_s_* = −0.456 [*P* < 0.001]; Sat-qPCR versus age [*n* = 80], *r_s_* = −0.647 [*P* < 0.001]; kDNA-qPCR versus age [*n* = 89], *r_s_* = −0.583 [*P* < 0.001]).

### Application of Sat-TcLAMP for monitoring parasite loads.

**FIG 9:**
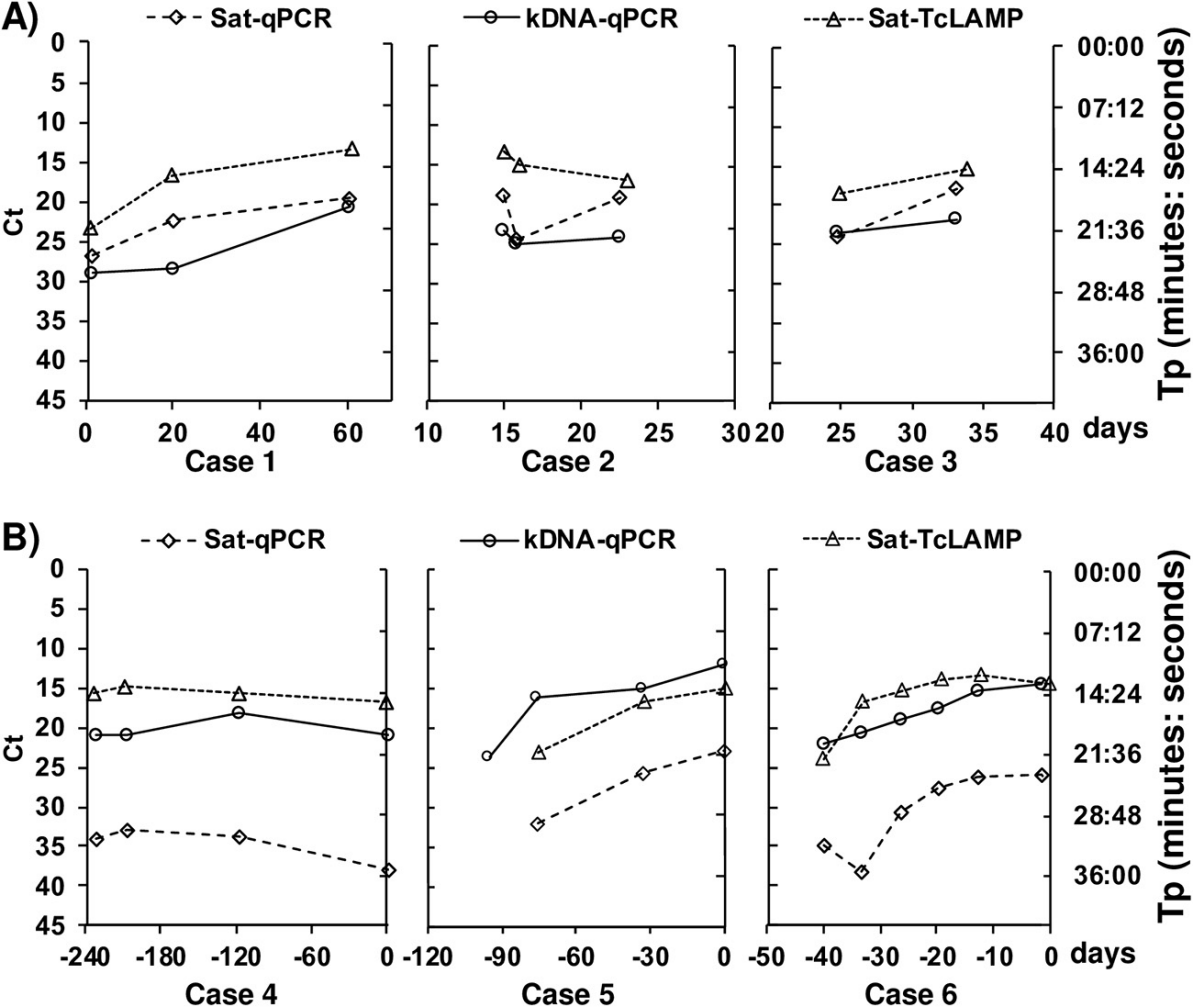
Parasitemia monitoring in terms of *C_T_* and *T_p_* values. Low *C_T_* values represent high levels of parasitemia. (A) Congenital infection. The horizontal axis represents days of life, with zero being the birth date. (B) Imported chronic infection. The horizontal axis represents days before treatment, with zero being the day of the beginning of treatment.

We included 6 cases (*n* = 23 samples), 3 with congenital infection (cases 1, 2, and 3) and 3 others with imported chronic infection (cases 4, 5, and 6) (Fig. 9). Due to different circumstances, they could not receive immediate treatment. In cases 1, 2, and 3, trypanocide treatment was started late due to inconveniences in acquiring the medication and changes in medical appointments. In cases 4, 5, and 6, the trypanocide drug was prescribed once the treatments for their other pathologies had finished or it could be combined with them. Therefore, the parasitemia levels were monitored at random times until the trypanocide treatment was initiated. Both kDNA tests, kDNA-cPCR and kDNA-qPCR, yielded positive results in the same number of samples (*n* = 22), whereas Sat-DNA tests, Sat-TcLAMP and Sat-qPCR, returned a negative result in 1 of them, just in the sample collected at the initial time of follow-up from case 5 (Fig. 9B). For case 6, the initial sample tested negative by all molecular techniques. Despite these differences, linear trends showed similar patterns for all quantitative approaches.

## DISCUSSION

In previous work (14), we studied the potential of Sat-TcLAMP for the diagnosis of congenital and chronic T. cruzi infection, and we showed that the performance of Sat-TcLAMP was equivalent to that of Sat-qPCR. In samples from children younger than 9 months of age, its sensitivity, specificity, and accuracy rates were 100%, 94%, and 96%, respectively. For imported chronic infection, the sensitivity of Sat-TcLAMP was low but enabled parasite detection with the same performance as that of Sat-qPCR (14). In this complementary study on Sat-TcLAMP, we explored for the first time its quantitative potential by using Sat-qPCR and kDNA-qPCR as comparators. Interestingly, we were able to draw the trend of parasitemia in the affected population living in an area where the disease is not endemic (Fig. 8), which clearly resembles the natural evolution of T. cruzi infection. Furthermore, Sat-TcLAMP enables monitoring the changes/trends in parasitemia as well as qPCRs do (Fig. 9).

Years ago, conventional PCR targeting T. cruzi kDNA was widely used for the qualitative detection of parasitemia. kDNA-PCRs with similar sensitivities among T. cruzi DTUs represent a clear advantage; however, in areas where Trypanosoma rangeli circulates, cross-amplification is considered a weakness (16). As T. rangeli does not circulate in Spain, this limitation is negligible. In chronic infection, our results and those reported in other studies show that kDNA-based tests have a higher sensitivity than Sat-DNA tests (Fig. 3) (16–18). This fact should be considered in posttreatment follow-up.

Currently, Sat-qPCR is the tool of choice for the quantification of T. cruzi parasitemia. However, due to the high index of copy number variation, the main drawback of using Sat-DNA as a target is that the sensitivity of the technique depends on the T. cruzi genotypes (16, 19, 20); e.g., in regions where TcI is predominant, parasite load estimations should be performed using a standard curve based on this DTU (16). In Spain, most T. cruzi-infected people come from Bolivia, but CD is also diagnosed in individuals from Mexico, Central America, and other countries of South America (21). Studies about DTU identification in blood samples in Latin American migrants living in Barcelona showed that TcV was the predominant DTU, but mixed infections of TcII/TcVI and TcII/TcV/TcVI were also found at similar rates (21, 22). In Madrid, TcV was also predominant, but single infections of TcIV, TcII, and TcI were also found (23). These studies also showed different rates of samples in which T. cruzi identification was not determined due to the low levels of parasitemia. Studies in Bolivia (regions of Cochabamba and Tarija) identified TcV and TcVI in samples from mothers and their newborns (24). In another region of Bolivia (Santa Cruz), TcV was the only DTU found in samples from infants with parasite loads above 1 × 10^4^ parasites/mL, but those authors did not identify which DTU was affecting the mothers due to their low levels of parasitemia (25). In our study, we did not identify which DTU was affecting each case, in some cases due to low levels of parasitemia (Fig. 7) and in others because of the lack of enough sample to perform all of the multilocus conventional PCRs required for genotyping (26). As the sociodemographic characteristics of our cases are like those reported in other studies in Spain, we assume that the frequencies of the different DTUs would be similar. A further factor to be considered is that TcI has a Sat-DNA copy number 1 logarithmic order lower than those for TcII, TcIV, and TcV, and this difference increases to 2 logarithmic orders compared with TcIII and TcVI. Thus, the differences between estimations from Sat-DNA and kDNA tests could be an expected outcome taking into account the Sat-DNA copy number variation among DTUs. As we do not know the real diversity of the T. cruzi populations affecting each patient, selecting the predominant DTU to build the standard curve does not seem to be the best option if we want to compare data from different regions with different circulating DTUs. To overcome this situation, the adoption of a single standard curve for the quantification of T. cruzi using a synthetic satellite unit DNA sequence was recently proposed (27). However, this strategy is also unable to estimate the parasitic load due to the impossibility of converting Sat-DNA copies into numbers of parasites without knowing which DTUs are infecting each patient. Therefore, a consensus on the best strategy to build a standard curve is needed. A potentially suitable candidate could be TcI because it has the lowest Sat-DNA copy number (27), it is the most ubiquitous DTU in areas of endemicity, and patients with TcI infection have been reported from North, Central, and South America (28, 29).

Currently, there is no consensus on the values at which parasitemia is considered low or high (30). In our previous work, the degree-of-parasitemia ranges were determined considering the LOD of a microhematocrit test according to data from previous studies by Vera-Ku et al. (LOD, 10,000 parasites/mL) (31) and Torrico et al. (LOD, 40 parasites/mL) (32). In this study, we also added a complementary LOD (500 parasites/mL) according to data described previously by Feilij et al. (33). Bearing these studies in mind, we considered values of ≤40 parasites/mL to be very low parasite loads, >40 to 500 parasites/mL to be low parasite loads, >500 to 10,000 parasites/mL to be moderate parasite loads, and >10,000 parasites/mL to be high parasite loads. Considering these ranges, and despite the differences in parasite load estimations, our data, like those from other groups, show that the parasitemia levels in congenital infections fluctuate from low to high levels (11, 20, 24, 34, 35). As shown in Fig. 8, if the infection is not treated, parasitemia decreases as a result of immune response control (6), fluctuating from very low to low parasite loads and even reaching undetectable levels. However, due to changes associated with aging, immunosuppression, or HIV coinfection, parasitemia could trend in different ways, even toward higher levels. It is noteworthy that our results, and those reported for another cohort of CD patients from Spain, are more comparable to those obtained in Bolivia, Argentina, and Brazil than to those reported in Colombia (Fig. 8B). This could be logical because people with CD living in Spain come from southern South America. In the last century, a low frequency of or no TcI was reported in blood samples from chronic CD cases; thus, TcI was considered innocuous. However, studies targeted at chronic CD patients with coinfection by HIV or with immunosuppression by a heart transplant showed that TcI was present in tissue samples (36, 37). In some circumstances, the tissue tropism of TcI seems to be higher than those of the rest of the DTUs, which could partly explain the low degree of parasitemia as a characteristic of immunocompetent patients from Colombia, even with acute infection, where 25 to 75% percentiles were 1.7 to 12.0 parasites/mL (38). In addition, in patients with multiple DTU infections, tropism differences were observed (36, 37). These factors could cause discrepancies in chronic infection in both qualitative and quantitative terms. In our study, the use of Sat-DNA did not detect parasitemia in chronic cases at the same proportion as that with kDNA, and estimations of parasitemia levels were lower than those estimated using kDNA. These differences have no clinical impact yet, but currently, most clinical trials are based exclusively on Sat-qPCR. Given the multiplexing capabilities that real-time PCR offers, perhaps both targets (Sat-DNA and kDNA) could be set up in the same reaction to make qPCR more cost-effective and reliable to quantify T. cruzi parasitemia, an approach used previously in Italy (39).

We identify two limitations of our work. The first limitation was that the LOD of Sat-TcLAMP has not been studied in depth. Previously, other authors described that the analytical sensitivities were similar between different DTUs (18), but considering the variability in the Sat-DNA copy numbers and discrepancies in samples with low parasite loads (Fig. 7 and Fig. 8A), we need to gain knowledge on this subject. Currently, there is no recommendation on how to construct the standard curve for Sat-TcLAMP because it has not been used previously for quantification. We decided to use all data that we had to compensate for the lack of an extensive characterization of the quantification limits. Therefore, we built the standard curve using the *T_p_* values from the analytical sensitivity experiments for TcI and TcV. With a suitable standard curve, the parasitemia level estimations by Sat-TcLAMP will probably improve. As the correlation between *C_T_* and *T_p_* values is notable (Fig. 4 and 9; see also Table S1 in the supplemental material), Sat-TcLAMP allowed the monitoring of parasitemia levels like qPCR, and thus, they can be used interchangeably for the early detection of the risk of reactivation in chronic infection (Fig. 9); similar findings were described previously by Muñoz-Calderón et al. (40) but in qualitative terms.

The second limitation is the retrospective design. Qualitatively, the Sat-DNA tests failed to detect parasitemia in some cases with chronic CD (Fig. 3). Quantitatively, Sat-qPCR showed lower levels of parasitemia than those determined by kDNA-qPCR (Fig. 7 and 8). In samples stored for long periods, kDNA could be more resistant to degradation than linear DNA due to the concatenated and supercoiled network structure of kDNA (24). To clarify this issue, a prospective comparison should be carried out, mainly for chronic infection, to determine its impact on posttreatment follow-up and parasitemia trends regarding the aging of patients. Curiously, although Sat-TcLAMP is also based on Sat-DNA, and the degradation of Sat-DNA could explain the results similar to those of Sat-qPCR in qualitative terms, Sat-TcLAMP detected parasite loads that were more equivalent to those determined by kDNA-qPCR. Considering the principle of LAMP (41), the formation of a stem-loop and cauliflower-like structures resembles the kDNA organization, which could explain the higher estimates of parasite load revealed by Sat-TcLAMP concerning Sat-qPCR.

Currently, treatment administration is not based on parasitemia levels; thus, differences between estimates do not affect the final clinical decision. However, it should be considered if a short scheme of treatment is finally introduced into clinical practice.

In conclusion, Sat-TcLAMP, Sat-qPCR, and kDNA-qPCR are similarly able to detect high parasitemia levels, meaning that any of the three methods could be used for the diagnosis of acute CD and the early detection of chronic CD reactivation. But with low parasite loads, which are characteristic of chronic CD, the methods have different performances, with kDNA-qPCR being the most sensitive. Therefore, to approach the detection and quantification of low levels of parasitemia, we propose combining Sat-DNA and kDNA as targets for qPCR methods because kDNA is present across all T. cruzi DTUs at similar numbers of copies and compensates for the high index of copy number variation of Sat-DNA. Also, when circulating DTUs are unknown, the standard curves for quantification should be built using a unique and same strain of TcI because this DTU has a limited copy number of Sat-DNA; thus, comparisons between studies could have the same constraint. Sat-TcLAMP and qPCRs showed that T. cruzi parasitemia in patients living in a country where the disease is not endemic resembles the natural evolution in areas of endemicity.

## MATERIALS AND METHODS

### Study design and samples.

This is a retrospective study based on a convenience sample of specimens from confirmed cases of CD who were diagnosed in Spain. A total of 173 samples collected from 156 patients were included: 159 samples were kept as guanidine-EDTA-blood (GEB), and 14 were kept as purified DNA. All GEB samples were included considering previous positive results by conventional kDNA PCR (kDNA-cPCR), except for two that were negative. One was previously positive by Sat-TcLAMP (14), and the other one was the initial sample from case 6, which is addressed further below. The 14 purified DNA samples were included considering previous positive results by Sat-qPCR.

Groups according to CD condition are shown in Fig. 1. The congenital infection group was composed of 39 cases (Fig. 1A), all of whom were born in Spain and had not traveled to countries of endemicity; 29 children were diagnosed before 9 months of age, and 10 were diagnosed at between 1 and 5 years of age. In children younger than 9 months old, CD was defined by positive results using parasitological and PCR tests; in older children, T. cruzi infection was defined by positive results using two serological tests and PCR. The chronic CD group was composed of 116 patients (Fig. 1B), all of whom were born in an area of endemicity, with positive CD serology (two serological tests) and ages of between 10 and 65 years. The group of individual cases in follow-up included samples from 3 congenital cases (cases 1, 2, and 3) and 3 chronic cases (cases 4, 5, and 6) who were in follow-up to monitor changes in parasitemia levels before treatment (*n* = 23), with samples being collected at random times according to clinical needs (Fig. 1C). In cases 1 and 2, three samples were collected from each patient at 3 different times (*T*_1_ to *T*_3_); in case 3, two samples were collected (*T*_1_ and *T*_2_); in cases 4 and 5, four samples were collected (*T*_1_ to *T*_4_), and in case 6, seven samples were collected (*T*_1_ to *T*_7_). The *T*_1_ samples from cases 1 to 5 were included in previous respective groups. The *T*_1_ sample from case 6 was not included in the chronic CD group because it was negative by kDNA-cPCR. Sociodemographic characteristics of cases were described previously by Flores-Chavez et al. (14).

In the congenital infection group, samples from children of different ages were included; 22 children were diagnosed during the acute phase, and 17 were diagnosed in an early chronic phase. Figure 2 shows the distribution of cases according to the phase of CD and other characteristics.

### DNA extraction methods.

Two different methods were used for DNA extraction. For kDNA-cPCR, DNA was purified according to methods described previously by Norman et al. (42), starting from 300 μL of GEB and resuspending the DNA in 75 μL of PCR-grade water (details are described in Annex S1 in the supplemental material). For quantitative molecular tests (Sat-qPCR and kDNA-qPCR) and Sat-TcLAMP, DNA was purified using the High Pure PCR template preparation kit (Roche Diagnostics, Germany) with minimal modifications, as described previously (14, 16, 43). Briefly, DNA was extracted from 300 μL of GEB and eluted in 100 μL of elution buffer supplied with the kit.

### Molecular tests.

Qualitative detection was performed by kDNA-cPCR according to methods described previously by Norman et al. (42) by using primers 121 and 122 and 10 μL of DNA and visualizing 25 μL of the amplified products on a 2% agarose gel with 0.1× GelRed nucleic acid gel stain (Biotum, USA) (details are described in Annex S1 in the supplemental material). All quantitative tests (Sat-qPCR, kDNA-qPCR, and Sat-TcLAMP) were performed using 5 μL of DNA. Sat-qPCR was run as described previously (16, 19, 44), with minor modifications, as described in previous work (14). kDNA-qPCR was carried out as described previously (24), with modifications. In a final volume of 20 μL, the reaction mixture was composed of 5 μL of purified DNA, 2 μL of 10× Quantimix master mix (Biotools B&M Labs SA, Spain), 1 μL of 20× Eva green dye (Biotum, USA), and 1 μL each of primers 121 and 122 (concentration of 200 ng/μL). The cycling conditions were 10 min at 95°C; 4 cycles at 95°C for 10 s, 68°C to 64°C for 15 s, and 72°C for 20 s (with a touchdown in the annealing temperature of 1 grade per cycle); and 45 cycles at 95°C for 10 s, 64°C for 15 s, and 72°C for 20 s using in a Rotor-Gene 6000 thermocycler (Corbett, Australia, and Qiagen, Germany). DNA melting curve and quantification analyses were performed to assess the amplification specificity (melting temperature, 82°C ± 1.5°C) and determine the cycle threshold (*C_T_*) values, respectively. Sat-TcLAMP was performed according to the instructions of the manufacturer, using a portable real-time fluorimeter (Genie III; OptiGene, UK). Briefly, 5 μL of DNA per reaction mixture was used, and amplification conditions were set at 95°C for 5 min, followed by 40 min at 65°C. Results provided by the fluorimeter were displayed at the end of the reaction as the time to positivity (*T_p_*) in minutes and seconds. These values were used to estimate parasitemia levels by Sat-TcLAMP.

### Standard curves.

For qPCRs, a standard curve was built by the amplification of eight 10-fold serial dilutions of T. cruzi genomic DNA from T. cruzi discrete typing unit (DTU) TcV, starting from 1 ng/μL DNA equivalents to 10^7^ parasite equivalents/mL, since we assumed 100 fg DNA to be equivalent to 1 parasite (15), and TcV was chosen considering the origin of the patients (22, 23). T. cruzi DNA was spiked into a pooled solution of human genomic DNA from uninfected individuals. For Sat-TcLAMP, the same serial dilutions of TcV as well as 10-fold serial dilutions of genomic DNA of T. cruzi DTU TcI were analyzed. As Sat-TcLAMP has not been used previously for quantification, there are no recommendations for the preparation of the standard curve. For this reason, the *T_p_* values from both analytical sensitivity experiments were used to build the standard curve for Sat-TcLAMP.

### Data analysis.

Multiple comparisons using nonparametric Cochran’s *Q* test were performed to conduct a qualitative analysis. Correlations between qPCR *C_T_* and Sat-TcLAMP *T_p_* values were evaluated by Spearman’s rho (*r_s_*) test. The parasitemia levels were estimated using standard curves and linear regression. Concordance between estimations was evaluated using Bland-Altman plots (45) plus linear regression, whereas differences between median values were assessed by multiple comparisons using the nonparametric Friedman test. The correlation between parasitemia levels and patient age at the time of diagnosis was determined by Spearman’s rho and multiple-regression analyses. The potential of Sat-TcLAMP to monitor parasitemia during the follow-up of individual cases was visualized by linear trend comparisons. All statistical analyses were performed using IBM SPSS Statistics v27.

### Ethical clearance.

The use of samples was approved by the ethics committees of each institution: the Research Ethics Committee of ISCIII, reference number CEI PI17_2011; the Clinical Research Ethics Committee of HSCSP, reference number IIBSP-CHA-2013-33 and CEIC number 53/2013; and the Research Ethics Committee of the Universitat de Barcelona, Institutional Review Board approval number IRB00003099. As this study was retrospective, formal consent was not requested from all patients. The recommendations of the respective ethics committees that consented to the use of these samples for research were followed, ensuring the confidentiality of patients by anonymizing their data, in compliance with current legislation. All samples were residual specimens from the diagnostic routine and were kept in sample collections after anonymization.
