# Supplementary material for: Parasitemia Levels in Trypanosoma cruzi Infection in Spain, an Area Where the Disease Is Not Endemic: Trends by Different Molecular Approaches
Source: Microbiol Spectr. 2022 Oct 3;10(5):e02628-22. doi: 10.1128/spectrum.02628-22 (PMC9603785; doi:10.1128/spectrum.02628-22)
Supplement: Supplemental file 1 — Supplemental material. Download spectrum.02628-22-s0001.pdf, PDF file, 0.2 MB [file spectrum.02628-22-s0001.pdf]

SUPPLEMENTAL MATERIAL

Annex 1. Description of Blood preparation and DNA Extraction for conventional kDNA-PCR (kDNA-cPCR)

Blood samples were collected and mixed with the same volume of 6 M guanidine-HCl and 0.2 M EDTA, pH 8.0. This sample was mixed on a shaker overnight at room temperature, after which it was boiled at 100 ºC for 15 minutes. Two aliquots of 300 μl were mixed with 200 μl of 100 mM NaCl, 1 mM EDTA, 10 mM Tris, pH 8, 50 ml of 10% SDS and 2 μl of 50 mg/ ml proteinase K, and incubated at 56 ºC for 1 hour. After incubation, 150 μl of 10% Chellex 100 (BioRad) were added in the same microtube and incubated at 60 ºC for 30 minutes, and then at 100 ºC for 10 minutes. The supernatant containing DNA was separated by centrifugation at 13,000 g for 10 minutes at room temperature. DNA was precipitated with 650 μl of isopropanol and 2 μl of 20 mg/ml glycogen by centrifugation at 13,000 g for 5 minutes at 4 ºC. The sediment was washed by centrifugation in 70% ethanol as before. Positive and negative controls in duplicate were included for each batch of 10 samples to monitor the risk of contamination. The limit of detection of this procedure is 1–0.1 parasites/ml (when the volume of the blood sample mixed with guanidine buffer is 10 ml). kDNA-cPCR amplification was performed in a total volume of 75 μl containing 10 mM Tris- HCl, pH 8.3, 50 mM KCl, 2 mM $MgCl_2$ and 0.2 mM of dNTPs, 0.5 μl of Taq DNA polymerase (Roche), 200 ng of 121 (5'- AAATAATGTACGGGKGAGATGCATG A-3') and 122 (5'-GGTTCGATTGGGGTTGGTGTAATATA-3') primers for *T. cruzi* detection, 5.6 pmol of REV (5'- GACGGTATCTGATCGTCTTC-3') and 1.1 pmol of HUF (5'-GAGCCGCCTGGATACCGC-3') primers for inhibition monitoring, and 10 μl of DNA of each sample or controls. The temperature profile was as follows: 95 ºC for 1 minute of denaturation with a longer initial time of 5 minutes at 95 ºC, 64 ºC for 1 minute for primer annealing and 72 ºC

for 1 minute for extension (35 cycles), with a final extension step at 72 ºC for 10 minutes. Positive and negative controls, in duplicate, were included for each amplification protocol. The analytical sensitivity is 0.01–0.001 parasites/reaction. The amplified products (25 µL) were visualized by electrophoresis on a 2% agarose gel with 0.1x GelRed™ Nucleic Acid Gel Stain (Biotum, USA). In the positive samples, an amplified product of 330 bp was observed. The PCR was valid when the controls were amplified correctly. DNA extraction was also valid when extraction controls were amplified correctly. The result of each sample was valid when both duplicates yielded the same result and when these were in accordance with the results of serological tests. When any of these criteria failed or the inhibition became evident, the whole procedure and serology were repeated.

Table S1. Coefficients of the Spearman's Rho and the confidence intervals

		N	Spearman's Rho	95% CI		p-value
**Total cases**	Sat-qPCR Ct *vs* kDNA-qPCR Ct	121	0.676	0.562	0.765	< 0.001
	Sat-qPCR Ct *vs* Sat-TcLAMP Tp	114	0.593	0.455	0.703	< 0.001
	kDNA-qPCR Ct *vs* Sat-TcLAMP Tp	116	0.644	0.519	0.742	< 0.001
**Cases with congenital infection**	Sat-qPCR Ct *vs* kDNA-qPCR Ct	39	0.831	0.694	0.91	< 0.001
	Sat-qPCR Ct *vs* Sat-TcLAMP Tp	38	0.653	0.413	0.808	< 0.001
	kDNA-qPCR Ct *vs* Sat-TcLAMP Tp	38	0.719	0.511	0.847	< 0.001
**Cases with imported chronic infection**	Sat-qPCR Ct *vs* kDNA-qPCR Ct	82	0.482	0.290	0.637	< 0.001
	Sat-qPCR Ct *vs* Sat-TcLAMP Tp	76	0.458	0.253	0.624	< 0.001
	kDNA-qPCR Ct *vs* Sat-TcLAMP Tp	78	0.527	0.339	0.674	< 0.001

Sat, satellite DNA. kDNA, variable region of the kinetoplast DNA minicircle. Ct, cycle threshold. Tp, time to positivity.

Table S2. Comparison of the parasite load according to molecular test

Category	Parameter	Sat-qPCR	kDNA-qPCR	Sat-TcLAMP	Friedman test (p value)
**0 to 65 yr**	Median (Q1 - Q3)	18 (2 – $6x10^2$)	40 (9 – $2x10^2$)	425 (33- $3x10^3$)	44.2
n = 110	Min - Max	0.1 – $5x10^5$	0.02 – $2x10^6$	0.8 – $2x10^5$	(<0.001)
**0 to 9 mo**	Median (Q1 - Q3)	3.10^3 (84 – $3x10^4$)	$2x10^2$ (80 – $9x10^3$)	$4x10^3$ (299 – $2x10^4$)	1.9
n = 29	Min - Max	0.3 – $5x10^5$	8 – $2x10^6$	3 – $2x10^5$	(0.381)
**> 9 mo to < 5 yr**	Median (Q1 - Q3)	6.10^2 (83 – $2x10^3$)	37 (26 – $3x10^2$)	597 (299 – $2x10^3$)	3.6
n = 9	Min - Max	4 – $6x10^3$	5 – $2x10^4$	157 – $4x10^4$	(0.169)
**10 to 65 yr**	Median (Q1 - Q3)	8 (0.7 - 32)	21 (6 – 74)	137 (18 – $1x10^3$)	54.2
n = 72	Min - Max	0.1 – $6x10^3$	0.02 – $2x10^4$	0.8 – $1x10^5$	(<0.001)

The null hypothesis 'the distributions of the parasite load by Sat-qPCR, kDNA-qPCR and Sat-TcLAMP were the same' was accepted only in congenital infection. mo, months; yr, years.
